# Effect of Maternal Factors and Fetomaternal Glucose Homeostasis on Birth Weight and Postnatal Growth

**DOI:** 10.4274/jcrpe.1914

**Published:** 2015-08-31

**Authors:** Öykü Özbörü Aşkan, Abdülkadir Bozaykut, Rabia Gönül Sezer, Tülay Güran, Abdullah Bereket

**Affiliations:** 1 Göztepe Medical Park Hospital, Clinic of Pediatrics, İstanbul, Turkey; 2 Zeynep Kamil Maternity and Children’s Diseases Training and Research Hospital, Clinic of Pediatrics, İstanbul, Turkey; 3 Marmara University Faculty of Medicine, Department of Pediatric Endocrinology, İstanbul, Turkey

**Keywords:** birth weight, glucose, insulin, large for gestational age, macrosomia

## Abstract

**Objective:**

It is important to identify the possible risk factors for the occurrence of large for gestational age (LGA) in newborns and to determine the effect of birth weight and metabolic parameters on subsequent growth. We aimed to determine the effects of maternal weight, weight gain during pregnancy, maternal hemoglobin A1c (HbA1c), C-peptide and insulin as well as cord C-peptide and insulin levels on birth weight and postnatal growth during the first two years of life.

**Methods:**

Healthy, non-diabetic mothers and term singleton newborns were included in this prospective case-control cohort study. Fasting maternal glucose, HbA1c, C-peptide and insulin levels were studied. Cord blood was analyzed for C-peptide and insulin. At birth, newborns were divided into two groups according to birth size: LGA and appropriate for GA (AGA). Infants were followed at six-month intervals for two years and their length and weight were recorded.

**Results:**

Forty LGA and 43 AGA infants were included in the study. Birth weight standard deviation score (SDS) was positively correlated with maternal body mass index (BMI) before delivery (r=0.2, p=0.04) and with weight gain during pregnancy (r=0.2, p=0.04). In multivariate analyses, the strongest association with macrosomia was a maternal C-peptide level >3.85 ng/mL (OR=20). Although the LGA group showed decreased growth by the 6-month of follow-up, the differences between the LGA and AGA groups in weight and length SDS persisted over the 2 years of follow-up.

**Conclusion:**

The control of maternal BMI and prevention of overt weight gain during pregnancy may prevent excessive birth weight. The effect of the in utero metabolic environment on the weight and length SDS of infants born LGA persists until at least two years of age.

## INTRODUCTION

Fetal overgrowth or macrosomia can be influenced by maternal diabetes, maternal obesity and excessive maternal weight gain during pregnancy (1,2,3). Adverse metabolic outcomes such as hyperglycemia, hyperinsulinemia and insulin resistance (IR) are common in obesity and gestational diabetes mellitus (GDM) (1,2). Maternal hyperglycemia stimulates fetal pancreatic hypertrophy and increase in fetal growth factors such as fetal insulin and insulin-like growth factor-1 (IGF-1) and leads to increased birth weight (3,4). Fuel-mediated teratogenesis and perinatal metabolic programming are important considerations concerning the effect of the maternal metabolic environment on the developing fetus (3).

Studies have shown that, as is the case for infants of women diagnosed with GDM, macrosomia is also common in infants born to non-diabetic obese women (1,2,3,5). Maternal body mass index (BMI) correlates with the incidence of large for gestational age (LGA) birth (2,6). In a study of women with GDM, pre-pregnancy BMI, weight gain before and after treatment and the treatment modality were all identified as predictors of infants being LGA, whereas maternal hemoglobin A1c (HbA1c) was not (6). Furthermore, the in utero obesogenic environment and epigenetic changes in the feto-placental unit may have long-term consequences for the offspring (3,7). In experimental studies on animals (8,9), maternal consumption of a high-fat diet prior to conception or maternal status as overweight lead to increased hyperinsulinemia, obesity, IR, hypertension, adiposity and endothelial dysfunction in the offspring. Additionally, these data suggested that maternal obesity leads to fetal programming, which can result in obesity in adulthood despite normal birth weight (8,9). Macrosomia may be associated with congenital anomalies, stillbirth or spontaneous abortion and children with a high birth weight have a greater risk of developing hypertension, obesity, cardiovascular dysfunction, metabolic syndrome and childhood-onset type 2 diabetes mellitus (DM) (3,10). In a study including 60 healthy women, maternal BMI early in pregnancy correlated with cord insulin and cord C-peptide levels and maternal BMI was suggested to be a predictor of fetal hyperinsulinemia (11). Cord blood C-peptide and insulin levels, maternal glucose and triglycerides levels, maternal BMI and homeostasis model assessment of IR (HOMA-IR) index have been reported as the main parameters which positively correlate with birth weight (1,2,3,4,11,12,13,14,15). However, it is difficult to compare the results of previous studies because of their different inclusion criteria, diverse glucometabolic parameters, use of different percentile charts and various time points of parameter measurement during pregnancies as well as ethnic and genetic anthropometric differences (1,3,4,6). The assessment of possible risk factors for the development of LGA newborns and of the effect of birth weight and metabolic parameters on growth during infancy is important.

The purpose of this study was to determine some of the fetomaternal variables that may potentially influence birth weight and postnatal growth during the first two years of life.

## METHODS

This was a prospective case-control cohort study. Our sample included healthy, non-diabetic mothers who gave consent to participate in the study together with their term singleton newborns. The newborns were in stable cardiopulmonary state in the delivery room.

A physical examination was conducted on every mother prior to delivery, at which time, blood pressure and maternal weight and height were measured. BMI was calculated as kg/m2. Pre-pregnancy BMI values were obtained from files and were classified based on the following World Health Organization (WHO) categories: obesity BMI ≥30 kg/m2; overweight 25-29.9 kg/m2; normal or underweight <25 kg/m2 (16). BMI prior to delivery was classified as follows: obesity ≥33 kg/m2, overweight 28.5-32.9 kg/m2 and normal or underweight ≤28.4 kg/m2 (7). Before a planned cesarean section, fasting blood samples were collected from each mother for determination of maternal glucose, HbA1c, C-peptide and insulin levels. Cord blood was collected from the placental side after the umbilical cord was clamped and analyzed for C-peptide and insulin. All blood samples were sent for processing immediately. Plasma C-peptide and insulin concentrations were measured by chemiluminescence on the Immulite 2000 (Siemens AG, Germany) and HbA1c was measured by an immunoturbidometric assay system (Cobas Integra 800, Roche Diagnostics GmbH, Germany). Serum C-peptide and insulin levels higher than 3.85 ng/mL and 25 µU/mL, respectively, were considered to indicate hyperinsulinemia. Data concerning pre-pregnancy weight, presence of postnatal respiratory distress, hospitalization in intensive care, Doppler ultrasonography (USG) results, type of delivery and previous test results from a 50-g oral glucose tolerance test at 24-32 weeks’ gestation were collected both from hospital records of prenatal control and from questionnaires. Diagnosis of GDM was made based on the 2010 recommendations of the International Association of Diabetes and Pregnancy Study Groups (17).

At birth, anthropometric measurements (weight, length) were obtained within 24 h of delivery. Birth weight was determined using a calibrated electronic scale with an accuracy of 2 g. In infants, length was measured with the infant in the supine position with a movable footboard (model 7725 baby scale; Soehnle Professional, Germany). Newborns were divided into two groups according to birth size. Specifically, birth weights above the 90th percentile or 2 standard deviation score (SDS) above the mean for gestational age were classified as LGA and birth weights between the 10th and 90th percentiles were classified as appropriate-for-gestational age (AGA) (Lubchenco growth charts) (18). Symmetry was evaluated by Rohrer’s ponderal index (PI= birth weight (g)/birth length (cm) 3×100), where infants with 2.25<PI<3.1 were considered symmetric. Each baby was examined for postnatal hypoglycemia 6 h after birth. Clinical neonatal hypoglycemia was defined as having a glucose value <47 mg/dL in the first 24 h after birth. Infants were followed at six-month intervals for two years, during which period, length and weight were recorded. The WHO Anthro (version 3.2.2, January 2011) and macros were used to calculate birth anthropometric SDS values.

Exclusion criteria were any medical or obstetric condition likely to affect birth weight such as endocrine disorders, a history of pre-pregnancy diabetes or any other chronic diseases (e.g. chronic renal disease, anemia, heart disease), multiple pregnancy, drug intake that interferes with glucose metabolism in the mother, gestational age ≤37 weeks, presence of congenital malformations and newborns resuscitated in the delivery room.

Written informed consent was obtained from the participants and the study was approved by the local ethics committee of Zeynep Kamil Maternity and Children’s Diseases Training and Research Hospital.

### Statistical Analysis

Statistical analyses were performed using SPSS version 17 (IBM SPSS Statistics, Chicago, IL). T-tests were used to analyze continuous variables. Categorical variables were converted to percentages and analyzed using the χ2 test. Pearson correlations were calculated to examine the relationships between maternal and infant measures. Logistical regression analysis was conducted to identify variables that were predictive of an infant being born ≥4000 g (dependent variable). The variables included in the regression were as follows: maternal age, gestational BMI, maternal height, infant gender, maternal hyperglycemia, hyperinsulinemia, a previous history of a macrosomic newborn and the current presence of hypertension or GDM. Determination of the best model was based on the Nagelkerke R2 value. A p-value<0.05 was considered statistically significant.

## RESULTS

A total of 83 newborns were included in this study: 40 LGA and 43 AGA infants. Median maternal age was 28 years. Average pre-pregnancy BMI was 26±0.6 kg/m2. Obesity was present in 19 mothers (22.9%). The 19 obese mothers delivered 8 LGA and 11 AGA babies, whereas non-obese mothers delivered 32 LGA and 32 AGA babies. GDM was present in 12 mothers, including 7 of the 19 obese mothers (37%) and 5 of 64 (8%) non-obese mothers. GDM mothers delivered 4 LGA and 8 AGA babies. Ninety percent of the LGA babies were born to non-GDM mothers. Insulin levels could be measured in 80 of 83 pregnancies. Monitoring of growth was conducted for two years in 17 of the 40 LGA newborns (42.5%) and 17 of the 43 AGA newborns (39.5%).

### Comparisons Between Appropriate for Gestational Age and Large for Gestational Age Groups

There were more asymmetric newborns in the LGA group (PI>3.1), with 40% and 9.3% in the LGA and AGA groups, respectively (p=0.004). The average maternal age was significantly lower in the LGA group (p=0.005). The neonatal and maternal characteristics as well as the glucometabolic parameters of the AGA and LGA groups in this study are presented in Table 1. Analyses revealed no significant differences between the AGA and LGA groups in occurrence of postnatal respiratory distress (p=0.3), hospitalization in intensive care (p=0.9), or in oral glucose tolerance test results (p=0.4), Doppler USG results (p=0.9) or in type of delivery (p=0.06).

### Correlations Between Maternal and Infant Glucometabolic Variables

Birth weight SDS was positively correlated with maternal BMI before delivery (r=0.2, p=0.04) and weight gain during pregnancy (r=0.2, p=0.04) but not with pre-pregnancy BMI (r=0.1, p=0.3). Maternal HbA1c (r=0.2, p=0.02) and cord insulin concentrations (r=0.2, p=0.01) were also found to be related to birth weight in each cohort. Post-pregnancy weights were positively correlated with C-peptide levels (r=0.2, p=0.04).

Subgroup analyses revealed that in the AGA group, there were no correlations between birth weight and HbA1c levels (r=0.3, p=0.8) or glucose levels at 6 h of life (r=0.2, p=0.08). However, in the LGA group, birth weight was positively correlated with maternal glucose (r=0.38, p<0.01) and HbA1c (r=0.45, p<0.005). Furthermore, cord C-peptide was positively correlated with maternal insulin (r=0.49, p<0.005) and C-peptide (r=0.33, p<0.05) and negatively correlated with neonatal glucose levels at 6 h of life (r=-0.46, p<0.005). Maternal systolic blood pressure was significantly correlated with maternal C-peptide (r=0.2, p=0.02), pre-pregnancy BMI (r=0.2, p=0.02) and post-pregnancy BMI (p=0.02).

Multivariate analyses showed that the presence of GDM increased macrosomia risk with an OR of 2.7. The strongest association with macrosomia was a maternal C-peptide level >3.85 ng/mL (OR= 20) (Table 2).

### Postnatal Growth

**Figure 1:**
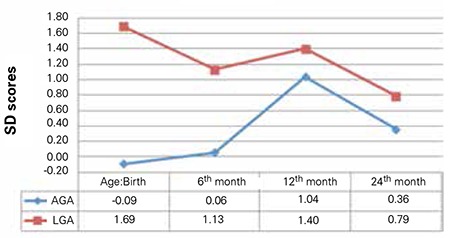
Comparison of the median weight standard deviation scores throughout infancy between appropriate-for-gestational age (AGA) and large-for-gestational age (LGA) newborns

**Figure 2:**
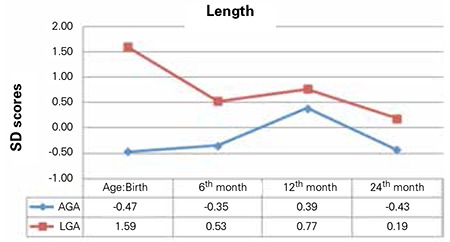
Comparison of the median length standard deviation scores throughout infancy between appropriate-for-gestational age (AGA) and large-for-gestational age (LGA) born newborns

The differences in weight and length SDS between AGA and LGA newborns persisted throughout infancy (Table 3). Analyses of the AGA group revealed that birth weight SDS correlated with weight SDS at 6, 12 and 24 months (r=0.7, p=0.001; r=0.6, p=0.002; r=0.6, p=0.007, respectively) (Figure 1). Birth length SDS correlated with length SDS at 6 and 24 months (r=0.5, p=0.04; r=0.5, p=0.02, respectively) but not at 12 months (r=0.4, p=0.05) (Figure 2).

The LGA group experienced a decrease in growth by 6 months of age. Birth weight SDS correlated with weight SDS at 24 months (r=0.5, p=0.01) but not at 6 or 12 months (r=0.3, p=0.1; r=0.4, p=0.06, respectively). Birth length SDS correlated with length SDS at 12 months (r=0.5, p=0.02) but not at 6 or 24 months (r=0.4, p=0.06; r=0.3, p=0.1, respectively) (Figures 1 and 2).

## DISCUSSION

In our study, birth weight correlated with higher maternal HbA1c, maternal BMI before delivery and excessive gestational weight gain. The variable with the strongest association with higher birth weight was maternal a C-peptide level >3.85 ng/mL. Furthermore, LGA newborns presented higher length and weight SDS throughout infancy than AGA newborns.

The prevalence of childhood obesity has shown a dramatic increase in many populations over the last decades (19). This phenomenon is expected to cause an increase in obesity-related health problems as well as on life expectancy, health care costs and national economies (19). Obviously, obesity links with excessive feeding and inactive life style. Maternofetal impacts should not be ignored in the development of obesity and postnatal programming.

Catalano et al (2) reported that the presence of obesity increases the incidence of macrosomic babies with an OR 1.68-1.73. Additionally, weight gain during pregnancy and pre-pregnancy BMI have been identified as the most influential characteristics affecting birth weight in the offspring of women without GDM (15,20,21,22). In women without GDM, it has been shown that the mean cord insulin and C-peptide levels are similar in AGA and LGA infants (21). Mitra et al (23) demonstrated that maternal BMI, gestational age, fasting serum insulin and random blood sugar showed significant positive correlations with birth weight (p<0.05); however, after regression analysis, only maternal BMI was identified as a significant predictor of neonatal birth weight. These observations suggest a direct influence of BMI and fat mass on increased maternal insulin secretion, which subsequently affects cord insulin and C-peptide levels and also infant birth weight (11,12,13,14). Higher IR has been demonstrated in LGA newborns (24) and non-obese prepubertal LGA children (25) than in AGA children. In recent years, improved maternal diabetes control and increased incidence of obesity have rendered maternal BMI an important determinant of birth weight. In our study, weight gain during pregnancy was higher in mothers of LGA newborns than in those of AGA, but this difference was not statistically significant. Birth weight SDS was positively correlated with maternal BMI before delivery but not with pre-pregnancy BMI.

Besides the mother’s weight status, her metabolic parameters also influence neonatal anthropometric outcome. In previous studies on a cohort of >20 000 newborns, 9.6% were reported to be macrosomic. The frequency of macrosomic babies was higher for mothers with higher maternal HbA1c and higher serum glucose concentrations (1,2). This observation was further supported by some other groups (1,11). Maternal C-peptide in mid-gestation (26) and higher HOMA-IR (13) are also reported to be associated with being born LGA.

Information about the metabolic outcomes of LGA newborns in their first years of life is limited. The EDEN Mother-Child Cohort Study Group examined the effects of fetal insulin and IGF-1 on postnatal growth. The results showed that an association existed between cord C-peptide and birth weight, but that after adjusting for IGF-1, this association disappeared. The authors suggested a role for IGF-1 on the casual path between C-peptide and birth weight (4). In this same cohort, fetal hyperinsulinemia has been shown to be associated with slower postnatal growth in girls in the 1st year of life but not in boys. In our study, length and weight SDSs in LGA newborns decreased by the 6th month but remained higher than those of AGA-born infants. Weaning from breastfeeding could be the reason for deceleration of growth around 6 months of age. This observation is consistent with the results reported by Çamurdan et al (27), who demonstrated that LGA infants showed a catch-down growth by the ninth month. On the other hand, introduction of complementary foods after the 6th month of life may have led to growth acceleration in AGA and LGA groups. Similar to our findings, Çamurdan et al (27) demonstrated variations of BMI from birth to the end of the 4th year of life; the BMI of LGA infants showed significantly higher values until age three years for LGA infants who were not breastfed. In a previous study, a two-year follow-up of LGA infants had demonstrated that birth weight is a significant predictor of weight at two years of age (22). Taken together, these results indicate that the effect of the in utero metabolic environment on the weight of infants is sustained in childhood.

Some limitations of our study should be noted. First, the study had a poor follow-up rate. In addition, we did not study maternal lipid profiles, including triglyceride, very low density lipoprotein cholesterol or IGF-1 concentrations, which may affect IR during pregnancy and the weight of the infants at birth. Because we were not able to repeat the blood tests at follow-up visits, we cannot interpret the long-term metabolic implications of birth weight.

In conclusion, the results of this study indicate that control of maternal BMI and prevention of overt weight gain during pregnancy may prevent excessive birth weight and also that the effect of the in utero metabolic environment on the weight and length SDS of infants born LGA is sustained until two years of age. Thus, fetomaternal influences should be considered as factors in the development of childhood obesity. These conclusions are in line with reports stating that negative metabolic outcomes may subsequently increase the chance of adulthood obesity up to 70% (28). Pre-pregnancy measures may break this vicious circle at an earliest setting.

## Figures and Tables

**Table 1:**
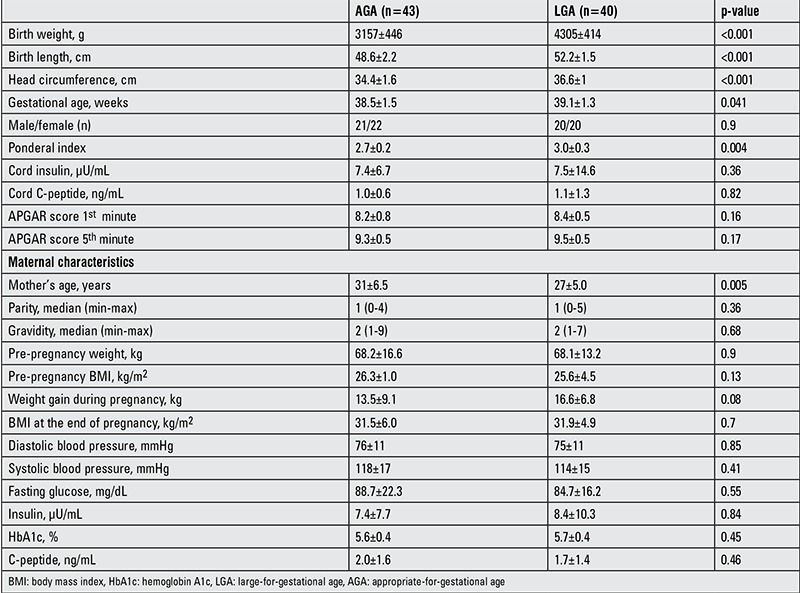
Characteristics of maternal and neonatal demographics in large-for-gestational age and appropriate-for-gestational age term newborns (results presented as mean ± SD unless otherwise noted)

**Table 2:**
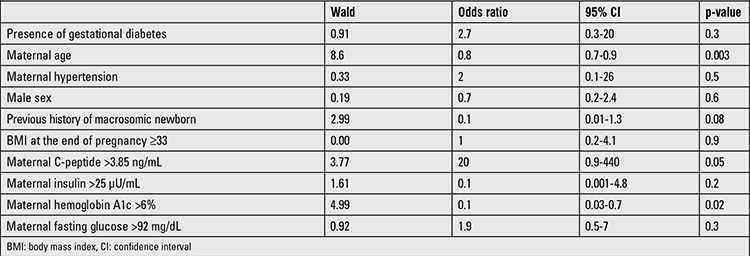
Results of a multivariate analysis of factors related to higher birth weight

**Table 3:**
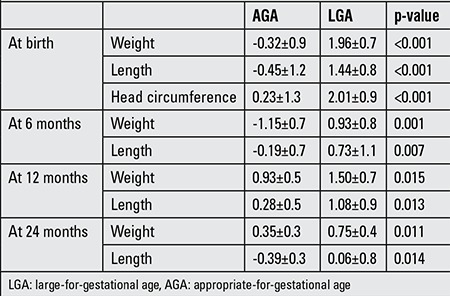
Comparison of SD scores for weight, length and head circumference at birth and in the first two years in appropriate-for-gestational age and large-for-gestational age newborns, (mean ± SD values)
